# Targeting the LPI/GPR55 Axis in MAFLD and MASH: Novel Insights, Therapeutic Strategies and Future Directions

**DOI:** 10.1111/liv.70576

**Published:** 2026-03-13

**Authors:** Jerome Lian, Ricky R Lareu, Mohan Patil, Marco Falasca

**Affiliations:** ^1^ Curtin Medical School, Curtin Medical Research Institute, Curtin University Perth Western Australia Australia; ^2^ Molecular Endocrinology and Pharmacology, Harry Perkins Institute of Medical Research and Centre for Medical Research, The University of Western Australia Nedlands Western Australia Australia; ^3^ Department of Medicine and Surgery University of Parma Parma Italy

**Keywords:** endocannabinoidome, hepatic steatosis, LPI/GPR55 axis, metabolic‐associated liver disease

## Abstract

Metabolic dysfunction‐associated fatty liver disease (MAFLD), recently redefined from non‐alcoholic fatty liver disease (NAFLD), highlights the central role of metabolic dysfunction in its pathophysiology. The L‐α‐lysophosphatidylinositol/G protein‐coupled receptor 55 (LPI/GPR55) axis, an element of the endocannabinoidome, has emerged as a key driver behind liver disease progression, leading to the progression of metabolic dysfunction associated steatohepatitis (MASH). Implicated in hepatic lipid accumulation, inflammation and fibrosis, this axis has detrimental effects in hepatocytes, Kupffer cells and hepatic stellate cells. Furthermore, recent evidence suggests that this axis induces *de novo* lipogenesis, promoting pro‐inflammatory cytokine production, leading to fibrosis and the transition toward a steatotic liver. The enzyme membrane‐bound O‐acyltransferase domain‐containing 7 (MBOAT7) modulates this axis by acylation of LPI, exacerbating hepatic steatosis and insulin resistance. Until recently, no pharmacologic treatments were approved for MAFLD. However, resmetirom received FDA approval in March 2024 for the treatment of MASH, and semaglutide (Wegovy) was granted accelerated FDA approval in August 2025 for MASH with moderate‐to‐advanced fibrosis. Additional agents such as tirzepatide and retatrutide remain in late‐stage clinical development. We propose that targeting the endocannabinoidome, specifically the LPI/GPR55 axis, represents a promising therapeutic strategy for liver disease. Previous attempts to target GPR55 therapeutically have involved small‐molecule agonists and phytocannabinoids with antagonistic activity. However, progress remains limited due to the context‐specific roles of GPR55 across different tissues and signalling pathways. As such, future strategies involving the LPI/GPR55 axis must focus on hepatic‐specific GPR55 modulation using selective ligands and advanced delivery systems, mitigating off‐target effects. This review elucidates the mechanistic role of the LPI/GPR55 axis, combining the role of MBOAT7 in the pathophysiology of metabolic‐associated liver disease.

## Summary

- LPI/GPR55 and MBOAT7/LPIAT1 pathways are linked to lipid signalling dysregulation in MAFLD/MASH.
- GPR55 has tissue‐specific roles: pro‐inflammatory in the liver and insulin‐sensitising in pancreatic β‐cells.
- MBOAT7 regulates LPI levels; its downregulation leads to pathological GPR55 activation.
- GPR55's dual effects and lack of tissue‐selective delivery challenge targeted therapy development but also present novel therapeutic opportunity by co‐targeting LPI/GPR55 and MBOAT7/LPIAT1.
- Combining pharmacological and gene therapies may advance treatment and deepen understanding of metabolic and immune dysregulation in MAFLD/MASH.

**Abbreviations**
2‐AG: 2‐arachidonoylglycerol
ACC: acetyl‐CoA carboxylase
AEA: anandamide
AKT: protein kinase B
CB1: canonical cannabinoid receptors 1
CB2: cannabinoid receptor 2
CBD: cannabidiol
DAMPs: damage‐associated molecular patterns
eCBome: endocannabinoidome
ECM: extracellular matrix
ECS: endocannabinoid system
ERK1/2: extracellular signal‐regulated kinases
FAS: fatty acid synthase
FXR: farnesoid X receptor
GLP‐1: glucagon‐like peptide 1
GPCRs: G‐protein coupled receptors
GPR119: G‐protein coupled receptor 119
GPR55: G‐protein coupled receptor 55
HCC: hepatocellular carcinoma
HSCs: hepatic stellate cells
IFN‐γ: interferon gamma
IL‐6: interleukin 6
LPI: L‐α‐lysophosphatidylinositol
LPIAT1: lysophosphatidylinositol acyltransferase 1
MAFLD: metabolic‐associated fatty liver disease
MASH: metabolic dysfunction‐associated steatohepatitis
MBOAT7: membrane‐bound‐O‐acyltransferase domain‐containing 7
MetS: metabolic syndrome
mTOR: mammalian target of rapamycin
NAFLD: nonalcoholic fatty liver disease
PI: phosphatidylinositols
PI3K: phosphatidylinositol 3‐Kinase
PUFAs: polyunsaturated fatty acids
ROCK: RhoA/Rho‐associated protein kinase
SCD1: stearoyl‐CoA desaturase‐1
SREBP‐1c: transcription factor sterol‐regulatory element binding protein‐1c
T2DM: type 2 diabetes mellitus
TGF‐β: transforming growth factor‐beta
THC: tetrahydrocannabinol
THCV: tetrahydrocannabivarin
TNF‐α: tumour necrosis factor alpha

## 1 Introduction

The term metabolic dysfunction‐associated fatty liver disease (MAFLD) represents a paradigm shift in the understanding of fatty liver disease. Formerly classified as non‐alcoholic fatty liver disease (NAFLD), the updated nomenclature of MAFLD emphasises the association of hepatic fat accumulation with other comorbid metabolic diseases, such as obesity and type 2 diabetes mellitus (T2DM) [1, 2]. While MAFLD is relatively benign, its progression to metabolic dysfunction–associated steatohepatitis (MASH) is associated with significant liver injury. This recently revised framework highlights the importance of the complex interplay between various metabolic diseases, in preference to the previous nomenclature focusing on the absence of excessive alcohol consumption. NAFLD, a term first coined in the 1980s, was defined as hepatic fat accumulation in individuals without significant alcohol‐related causes of liver disease. Serving its purpose for decades, it has recently come under scrutiny for its exclusionary nature, failing to encompass the complex role of metabolic dysfunction as a driving force in the progression of liver disease [3]. Because NAFLD classification hinges on excluding significant alcohol consumption, it groups individuals with metabolic syndrome (MetS) with those without MetS, even though their underlying pathophysiology can differ substantially.

MAFLD has now become the most prevalent chronic liver disease worldwide and is expected to affect approximately 33.5% of the global population by 2030 [4]. This increase is attributed to the rising rates of obesity, T2DM and closely related metabolic disorders. In the US, rates of MAFLD in the adult population have seen a prevalence of up to 39.1% [5]; meanwhile, in Australia, the prevalence of MAFLD is expected to increase from 5.5 million cases in 2019 to over 7 million by 2030, representing a 25% increase [6]. Despite the limited epidemiology of MAFLD in Europe, studies investigating a large cohort in the United Kingdom found that 38% of adult participants had MAFLD [7]. The prevalence of MAFLD varies by region, with higher rates observed in areas experiencing significant lifestyle and dietary changes [8]. However, a common theme observed globally is the rise in a sedentary lifestyle, coupled with foods that are high in saturated fats and sugar [9]. Namely, this can be observed in a Western‐style fast food diet, whereby foods high in saturated fats and sugar have caused the soaring rates of obesity, T2DM and MAFLD. For instance, MAFLD rates exceed 40% in North America [10] while Africa has an average rate of 13% [11]. Pharmacological strategies for metabolic dysfunction‐associated steatotic live disease (MASLD) and MASH are undergoing rapid evolution. In early 2024, Rezdiffra (resmetirom), a liver‐targeted thyroid hormone receptor β‐selective agonist, became the first agent to receive regulatory approval for the treatment of MASH (NCT03900429) [12]. More recently, Wegovy (semaglutide), a glucagone‐like peptide‐1 (GLP‐1) agonist, also achieved regulatory approval in this therapeutic domain (NCT04822181) [13]. In parallel, other incretin‐based agents, including tirzepatide (NCT04166773), survodutide (NCT04771273) and retatrutide (NCT04881760) are progressing through advanced stages of clinical development, highlighting a rapidly expanding therapeutic landscape [13, 14]. Besides such advancements, novel therapeutic avenues are being investigated, with the extended endocannabinoid system emerging as a promising target. The role of lipids extends beyond serving as the structural components of cell membranes, acting as potent signalling molecules that regulate key cellular processes such as inflammation, metabolism and cell proliferation. Dysregulation of lipid signalling pathways has been increasingly implicated in the pathogenesis of MAFLD, highlighting the importance of understanding specific lipid‐mediated axes in metabolic disease progression [15]. This review aims to summarise currently available information on the lysophosphatidylinositol/G protein‐coupled receptor 55 (LPI/GPR55) axis and the important role of membrane‐bound‐O‐acyltransferase domain‐containing 7 (MBOAT7), highlighting their potential as a therapeutic target for the treatment of MAFLD/MASH.

## 2 The Endocannabinoid System

### 2.1 Canonical ECS Receptors

The endocannabinoid system (ECS) has emerged as a key regulator of various physiological processes, namely lipid metabolism, glucose metabolism and appetite control [16]. The ECS encompasses endogenous ligands (endocannabinoids), receptors and enzymes that synthesise and degrade the ligands. Dysregulation of the ECS has been implicated in numerous metabolic disorders, including obesity, T2DM and MAFLD [17, 18]. The canonical cannabinoid receptors 1 (CB1) and cannabinoid receptor 2 (CB2) have been extensively studied for their significant roles in disease pathophysiology, in hopes of finding new therapeutics [19]. These G‐protein coupled receptors (GPCRs) are activated by endogenous ligands such as anandamide (AEA) and 2‐arachidonoylglycerol (2‐AG), as well as exogenous phytocannabinoids, including tetrahydrocannabinol (THC) and cannabidiol (CBD). Despite belonging to the same family of receptors, the modulatory effects of both CB1 and CB2 remain distinct. CB1 is most abundantly distributed in the brain, where it is highly expressed in the basal ganglia nuclei, cortex, hippocampus and cerebellum [20, 21]. High expression in the central nervous system correlates with the role of CB1 in appetite, motor function, memory, cognition and analgesia [22]. Overactivation of CB1 receptors has been found to be correlated with hyperphagia, obesity and metabolic disorders [23]. Conversely, CB2 localisation can be seen in peripheral organs with immune function, including the thymus, spleen and lung, in addition to macrophages and leukocytes [24, 25]. Despite the extensive studies conducted into the therapeutic potential of targeting these canonical receptors, there is still a lack of commercially available therapeutics that target the ECS. Firstly, the complex and ubiquitous nature of the ECS makes drug target specificity a challenge [26, 27]. Further complicated by the pleiotropic effect of the ECS, targeting a specific pathway without disrupting another has proven to be difficult, often presenting in the form of adverse effects [28]. At its peak in 2006, the drug Rimonabant, an anti‐obesity drug that antagonises the CB1 receptor, was hailed as a success in modulating the ECS [29]. However, the drug was quickly withdrawn 2 years later due to serious unwanted psychiatric side effects [30].

### 2.2 Extended Family of GPCRs, GPR18, GPR55 and GPR119

As research has uncovered a broader array of receptors, ligands and metabolic pathways beyond the classical ECS, the term endocannabinoidome (eCBome) has been introduced to reflect this expanded and functionally diverse network. Increasing evidence now suggests that modulating this extended family of ECS components may offer therapeutic advantages [31]. This extended family of receptors also exhibits endocannabinoid‐like receptor activity, with notable receptors relevant to metabolic disorders such as G‐protein coupled receptor 119 (GPR119) and GPR55. Indeed, the Class A orphan GPCRs GPR18, GPR55 and GPR119, each reported to respond to endogenous compounds analogous to cannabinoid ligands, have been collectively classified as a distinct group [32]. GPR18 has been implicated in the regulation of immune responses and inflammation, but emerging evidence also suggests a role in metabolic processes and liver function [33]. GPR119 plays a crucial role in the control of glucose and insulin sensitivity, signifying a potential therapeutic pathway for the treatment of glucose‐related metabolic disorders [34]. GPR55, previously thought to be a putative CB3 receptor, has been found to share limited homology to CB1 (13.5%) and CB2 (14.4%) [35]. Furthermore, GPR55 differs from CB1 and CB2 structurally by the lack of a cannabinoid binding pocket but rather has a binding pocket with many hydrophilic residues in its active conformation [16, 36, 37, 38, 39, 40]. The endogenous ligand for GPR55 is LPI, an endocannabinoid‐like molecule [41]. However, GPR55 has been found to also be sensitive to a range of endogenous and exogenous ligands like AEA and 2‐AG [42]. In addition, GPR55 has been shown to interact with various exogenous ligands, including cannabinoids. GPR55 is moderately to highly expressed in the liver, adipose tissue (especially white adipose) and immune cells, where it regulates lipid metabolism, insulin sensitivity and inflammatory responses. Its activation, primarily by ligands like LPI, influences hepatocyte and adipocyte function as well as immune cell migration and cytokine release [43]. The LPI/GPR55 axis has been known to play an oncogenic role, participating in cellular proliferation, migration, invasion and metastasis [44, 45, 46]. Recently, the LPI/GPR55 axis has garnered interest in liver disease, with emerging evidence suggesting the causal role of the LPI/GPR55 axis as a driver for MAFLD [47, 48]. The role of LPI, more specifically the subspecies Oleoyl‐LPI, has been found to be a potent activator of GPR119, stimulating the release of GLP‐1 [49]. Similar to GPR119, the distribution of GPR55 receptor activity can be found in key tissues relating to glucose homeostasis, whereby organs such as the pancreas, liver, GI tract, skeletal muscle and adipose tissue have significant GPR55 activity [50]. GPR55 primarily signals through the Gα13 protein, activating downstream signalling pathways such as RhoA/Rho‐associated protein kinase (ROCK) and extracellular signal‐regulated kinases (ERK1/2) [51]. Consequently, these downstream effectors are involved in cell proliferation, cytoskeletal remodelling and inflammatory responses, which are particularly important to metabolic regulation [43, 50].

## 3 Biosynthesis and Metabolic Control of Endogenous Lysophosphatidylinositol

Endogenous LPI is produced primarily through the hydrolysis of phosphatidylinositol (PI) by phospholipase A1 or A2 (PLA1/PLA2) enzymes in cellular membranes [41]. In the liver, hepatocytes and Kupffer cells represent major sources, although adipose tissue, immune cells and platelets also contribute to circulating LPI pools. Under normal physiological conditions, LPI levels are tightly regulated by a balance between phospholipase activity, lysophospholipase degradation and lipid transport and remodelling pathways within the endoplasmic reticulum and plasma membranes.

Metabolic and inflammatory stressors can disrupt this balance. In states of insulin resistance, obesity and hepatic lipotoxicity, increased phospholipase activation and oxidative membrane remodelling lead to elevated hepatic and plasma LPI levels. Pro‐inflammatory cytokines such as tumour necrosis factor alpha (TNF‐α) and interleukin 6 (IL‐6) further enhance phospholipase activity, while down‐regulation of lysophospholipases contributes to LPI accumulation. Circulating LPI levels are elevated in obesity and MASLD. LPI may exist in a variety of acyl‐chain forms, with 16:0, 18:0 and 20:4 being among the more prevalent species to exist [52, 53]. This highlights the importance of different LPI species and the divergent biological effects they exert [54]. In normal homeostatic conditions, plasma LPI ranges between 1 and 10 μM [53]. However, in MetS, these levels are elevated, positively correlating with body mass index, body fat percentage and insulin resistance [55, 56, 57]. Elevated levels of LPI are also correlated with the upregulation of GPR55 in adipocytes, thus linking the LPI/GPR55 axis to metabolic dysfunction [41]. Furthermore, LPI levels are also elevated in various cancer types, reinforcing its pathophysiological role [46, 58]. Recent studies have shown that circulating LPI concentrations correlate positively with hepatic steatosis, fibrosis stage and systemic metabolic markers, suggesting that endogenous LPI serves both as a metabolic stress signal and a ligand for GPR55 activation during MASLD progression [47]. Conversely, conditions that promote enhanced fatty acid oxidation or membrane lipid remodelling (e.g., caloric restriction, exercise or treatment with metabolic modulators such as GLP‐1 receptor agonists) may lower LPI levels [59]. These findings collectively support a model in which endogenous LPI acts as a dynamic lipid messenger, linking metabolic imbalance and inflammatory activation through GPR55 signalling in the liver. LPI can be produced by endothelial cells endogenously and has been demonstrated to activate GPR55 in human endothelial cells [60]. By modulating the potassium channels, LPI acts as an intracellular messenger through changes in calcium ions. Further in vitro studies have also shown that LPI‐GPR55 activation resulted in increased endothelial cell activation and suppressing autophagy, thereby exacerbating atherosclerotic lesions [61]. Further evidence reinforces the pathogenic characteristics of LPI, showing that LPI negatively impacts the motility of intraepithelial lymphocytes (IELs), causing susceptibility to intestinal injury [62]. The same study showed that GPR55‐deficient mice had the ability to recruit greater number of IELs, thus showing resistance to intestinal injury and showing stronger integrity of the gut epithelial barrier. The impact of disruptions to the gut epithelial barrier and subsequent translocation of microbial products in the pathophysiology of MASLD has been well documented [63, 64]. Likewise, a mice model of pancreatic cancer showed that GPR55 deficiency in tumour microenvironment resulted in increased CD3^+^/CD8^+^ T‐cell infiltration, leading to a reduction in tumour weight and volume [65]. In contrast, GPR55 activation by ligands other than LPI has has been shown to enhance proliferation in cholangiocarcinoma cell lines expressing functional GPR55 [66], further reinforcing the pathogenic role of GPR55 signalling.

## 4 The LPI/GPR55 Axis as a Driver of MAFLD

In the liver, GPR55 is expressed in hepatocytes, Kupffer cells and hepatic stellate cells (HSCs) [47]. Hepatic GPR55 expression has been found to influence lipid metabolism, inflammation and fibrosis, all of which are key processes in the pathogenesis of MAFLD [47]. Growing evidence suggests that overexpression of GPR55 in the liver is associated with the development of MAFLD and subsequent progression to MASH [47, 67, 68].

Several preclinical and translational studies have investigated the LPI/GPR55 axis in the setting of hepatic steatosis, inflammation and fibrogenesis. Table S1 summarises species, experimental approach, treatment (endogenous vs. exogenous), dosing (when reported) and whether sex‐specific analyses were performed. Experimental work in male C57BL/6J mice has shown that exogenous LPI administration exacerbates hepatic steatosis and worsens liver histology, supporting a pathogenic role for LPI in metabolic liver injury [47]. In complementary human studies, elevated hepatic GPR55 mRNA expression has been observed in individuals with obesity, and higher circulating concentrations of LPI species, particularly LPI 16:0 and LPI 18:1, have been reported in patients with steatohepatitis compared with those with simple steatosis. Additional mouse models have demonstrated that endogenous LPI levels rise in response to high‐fat diet feeding, with LPI 18:0 showing a significant increase relative to controls [69]. Together, these findings suggest that activation of GPR55 by LPI may be an important mechanistic driver of MASLD progression.

### 4.1 GPR55 in Hepatocytes

Hepatocytes are the principal parenchymal cells of the liver and are crucial in metabolic homeostasis maintenance. Responsible for the metabolism and storage of lipids, LPI‐induced GPR55 activation in hepatocytes often results in the disruption of lipid metabolism. Upregulation of GPR55 in hepatocytes correlates with the promotion of *de novo* lipogenesis through the upregulation of key lipogenic enzymes such as fatty acid synthase (FAS), Acetyl‐CoA carboxylase (ACC) and stearoyl‐CoA desaturase‐1 (SCD1). Overactivation of FAS resulting from LPI/GPR55 axis activation leads to an excess in the conversion of carbohydrates into fatty acids, significantly contributing to hepatic lipid accumulation [70]. Meanwhile, LPI/GPR55 axis activation of ACC enhances malonyl‐CoA production, driving fatty acid synthesis. Malonyl‐CoA also inhibits fatty acid oxidation by inhibiting carnitine palmitoyltransferase 1, further promoting lipid accumulation [71]. Studies have also shown that the downregulation of ACC reduced subsequent hepatic steatosis, independently of glucose homeostasis [72, 73, 74]. In a similar study, it was found that ACC knockout mice had increased total energy expenditure, lower fat mass and greater insulin sensitivity [75]. The LPI/GPR55 axis has also been shown to increase the expression of SCD1, responsible for increased monounsaturated fatty acid production, leading to increased triglyceride synthesis and lipid droplet formation [76]. The transcription factor sterol‐regulatory element binding protein‐1c (SREBP‐1c) is a key regulator of the aforementioned lipogenic enzymes, and its expression is enhanced by LPI/GPR55 signalling, amplifying the lipogenic pathways in hepatocytes to drive steatosis [69]. Ultimately, evidence suggests that the LPI/GPR55 axis activation shifts normal functioning hepatocytes toward a lipogenic phenotype, coupled with increased triglyceride accumulation to drive hepatic steatosis.

### 4.2 LPI/GPR55 in Kupffer Cells

**FIGURE 1:**
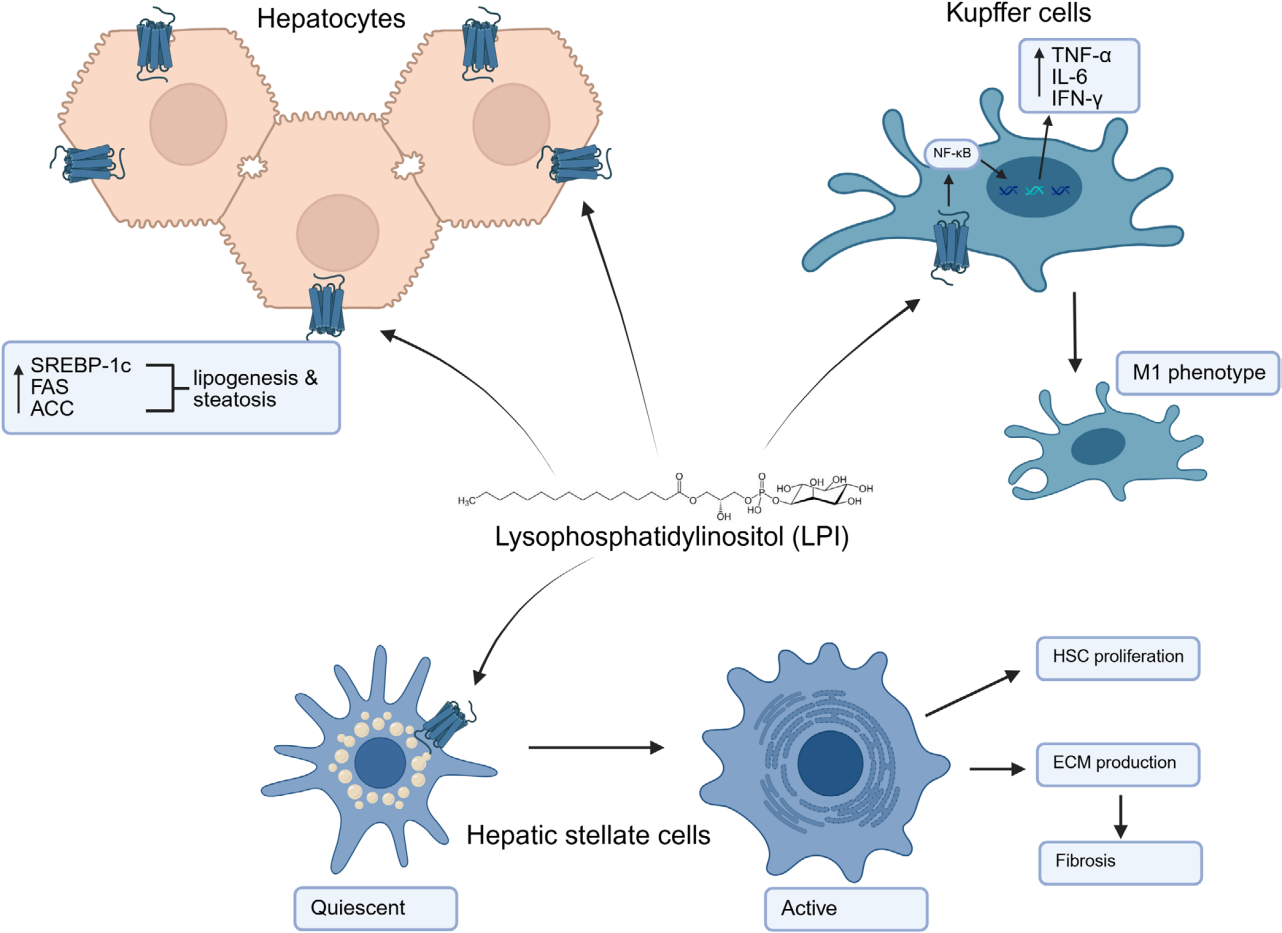
Pathogenic LPI/GPR55 signalling in the liver activates hepatic stellate cells, promotes extracellular matrix deposition, induces SREBP‐1c–mediated lipogenesis via ACC and FAS, and enhances inflammation through TNF‐α, IL‐6, and *IFN*‐γ. ACC, Acetyl‐CoA carboxylase; ECM, extracellular matrix; FAS, fatty acid synthase; HSC, hepatic stellate cells; IFN‐γ, interferon gamma; IL‐6, interleukin 6; SREBP‐1c, transcription factor sterol‐regulatory element binding protein‐1c; TNF‐α, tumour necrosis factor alpha. Created with BioRender.com.

Kupffer cells are the resident liver macrophages, whose activity impacts the pathophysiology of MAFLD by driving chronic low‐grade inflammation often characterised in early MAFLD stages [77]. LPI/GPR55 activation alters Kupffer cell behaviour, upregulating pro‐inflammatory cytokine production, TNF‐α, IL‐6 and interferon gamma (IFN‐γ) [47]. Furthermore, LPI in its albumin‐bound form was found to induce the release of TNF‐α and IL‐6 from macrophages [78]. While Kupffer cells exhibit functional plasticity, LPI/GPR55 activation skews their polarisation away from the anti‐inflammatory M2 macrophage phenotype toward the pro‐inflammatory M1 phenotype, as illustrated in Figure 1. In the pro‐inflammatory state, Kupffer cells can be characterised by high production of pro‐inflammatory cytokines, reactive oxygen species and nitric oxide [79]. Undoubtedly, oxidative stress remains a major driver of liver damage seen in advanced stages of MAFLD [80]. LPI/GPR55 activation of Kupffer cells significantly contributes to hepatocyte damage through a variety of means. Kupffer cells in this state enhance the production of reactive oxygen species, damaging hepatocytes and perpetuating inflammation [81]. In addition, damaged hepatocytes release damage‐associated molecular patterns (DAMPs), upregulating the activation of Kupffer cells and amplifying inflammatory signalling [82]. In the progression of MAFLD to MASH, a defining feature is the presence of hepatic fibrosis, driven by the activation of HSCs. LPI/GPR55 causes Kupffer cells to release pro‐fibrotic mediators, causing crosstalk with HSCs. TNF‐α and IL‐6 released by Kupffer cells stimulate HSC activation, leading to fibrosis through the overproduction of collagen [83]. Transforming growth factor‐beta (TGF‐β) and platelet‐derived growth factors released by Kupffer cells directly activate HSCs, illustrating the damaging effects of LPI/GPR55 activation in the liver [84].

### 4.3 LPI/GPR55 Activation of Hepatic Stellate Cells

Under pathological conditions such as chronic inflammation and excess lipid accumulation, activation of HSCs transforms their behaviour from vitamin A‐storing cells to myofibroblast‐like cells that secrete excessive extracellular matrix (ECM) proteins [85]. Consequently, this excess in ECM proteins such as collagen disrupts liver architecture and function, leading to portal hypertension, tissue hardening and the potential to progress toward a cirrhotic liver. LPI/GPR55 activation of HSCs has been increasingly recognised as a key modulator behind the fibrosis response, linking metabolic dysfunction, chronic inflammation and steatosis to liver fibrosis in MAFLD [48]. Moreover, LPI/GPR55 activation of HSCs enhances the expression of pro‐fibrotic transcription factors while also inhibiting the activity of matrix metalloproteinases, resulting in impaired ECM degradation. LPI/GPR55 may also worsen MAFLD by promoting HSC proliferation and migration to the liver injury site, thus amplifying the fibrotic response.

### 4.4 GPR55 in Other Cell Types

While functional work to date has largely focused on hepatocytes, Kupffer cells and HSCs, it is important to note that GPR55 is also expressed in other hepatic and non‐parenchymal cell populations such as biliary epithelial (cholangiocyte) cells (as shown in human cholangiocyte cell lines) [66], endothelial cells (as demonstrated in human aortic endothelial models) [86] and immune cell subsets including T‐cells, B‐cells and dendritic cells (mouse and human studies) [87]. These broader expression patterns underscore the possibility that GPR55 signalling may influence additional aspects of the hepatic microenvironment, such as sinusoidal endothelial function, biliary pathology or intra‐hepatic immune cell regulation, that remain under‐studied in the context of MASLD and MASH.

### 4.5 GPR55 in Activation in Extrahepatic Tissues

MASLD is tightly linked to MetS, including obesity, dyslipidaemia and insulin resistance. Consequently, GPR55 signalling in extrahepatic tissues may indirectly modulate hepatic outcomes. For example, white adipose tissue expresses GPR55, and preclinical studies indicate that GPR55 activation influences adipocyte lipolysis, inflammatory cytokine release and insulin sensitivity. Improvements in systemic metabolic parameters via extrahepatic GPR55 modulation could therefore secondarily reduce hepatic lipid accumulation and inflammation, complementing any direct hepatic effects.

These observations raise the question of whether therapeutic strategies should aim for liver‐specific GPR55 modulation or leverage systemic effects. Liver‐targeted delivery could maximise direct hepatocyte or stellate cell effects while minimising off‐target actions in other tissues, whereas systemic modulation might provide broader metabolic benefits but require careful consideration of safety and tissue‐specific signalling.

Another consideration is that overall hepatic expression of GPR55 is relatively low compared with other metabolic or inflammatory receptors. This suggests that direct hepatocyte‐mediated effects may be modest, emphasizing the potential importance of paracrine, immune‐cell‐mediated and extrahepatic mechanisms in modulating liver disease. Future pharmacologic approaches may need to balance direct hepatic receptor engagement with systemic metabolic modulation to achieve maximal therapeutic benefit in MASLD and MASH.

## 5 MBOAT7/LPIAT1 and MAFLD Progression

Membrane‐bound‐O‐acyltransferase domain‐containing 7 (MBOAT7), also known as lysophosphatidylinositol acyltransferase 1 (LPIAT1), is an enzyme that plays an integral role in lipid remodelling [88]. MBOAT7/LPIAT1 alters the composition of phosphatidylinositols by incorporating arachidonic acid into the nucleophilic substitution 2 position of LPI to form PI, thus maintaining a fundamental role in lipid homeostasis and signalling [89]. This acylation process also plays a critical role in maintaining membrane fluidity, supporting intracellular signalling and regulating lipid and triglyceride synthesis. Through the Lands cycle, MBOAT7/LPIAT1 governs the reacylation of lysophospholipids, and dysfunction of this enzyme has been linked to liver disease and other neurological disorders [89, 90, 91]. The first study to associate MBOAT7/LPIAT1 variants with liver disease was published in 2015 [92] and since then, emerging evidence suggests that downregulation of MBOAT7/LPIAT1 has been implicated in the pathogenesis of MAFLD and MASH. Genome‐wide association studies have identified genetic variants of MBOAT7/LPIAT1, most notably the single nucleotide polymorphism rs641738 C>T, as a risk factor for MAFLD pathogenesis [93]. This risk variant causes a loss of function to the normal MBOAT7/LPIAT1 gene, resulting in hepatic lipid accumulation, fibrosis progression and inflammation [94]. This notion of MBOAT7/LPIAT1 loss of function is further solidified by higher levels of circulating LPI found in individuals with MetS [43, 95, 96]. The localisation of MBOAT7/LPIAT1 to the endoplasmic reticulum is attributed to its importance in maintaining phospholipid composition and regulating key lipid signalling pathways, while also exerting a hepatoprotective effect against alcohol‐induced liver disease through regulation of lysosomal lipid homeostasis [97].

**FIGURE 2:**
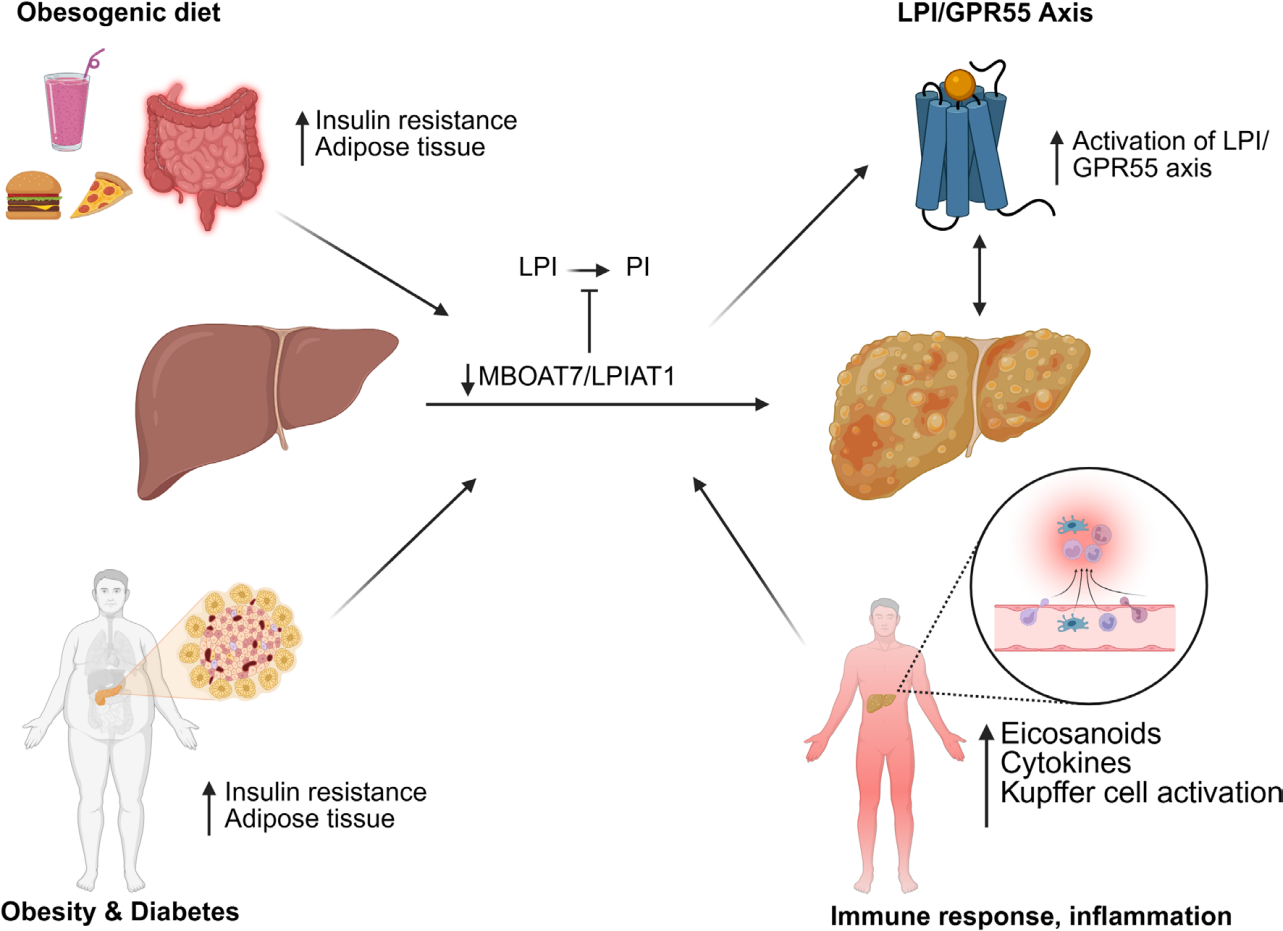
MBOAT7/LPIAT1 deficiency and pathogenic LPI/GPR55 signalling drive MAFLD and MASH by disrupting PI remodelling and promoting hepatic inflammation and steatosis. GPR55, G‐protein coupled receptor 55; LPI, L‐α‐lysophosphatidylinositol; LPIAT1, lysophosphatidylinositol acyltransferase 1; MAFLD, metabolic‐associated fatty liver disease; MASH, metabolic dysfunction‐associated steatohepatitis; MBOAT7, membrane‐bound O‐acyltransferase domain‐containing 7; PI, phosphatidylinositols. Created with BioRender.com.

In the liver, MBOAT7/LPIAT1 influences MAFLD/MASH progression in three primary mechanisms, summarised in Figure 2. Firstly, deficiencies in MBOAT7/LPIAT1 activity lead to impairments in phospholipid remodelling, causing changes in PI composition [98, 99, 100]. More importantly, the acyl chain attached to PI and its proper composition are crucial for maintaining membrane function and signalling [101]. Deficiencies in MBOAT7/LPIAT1 activity have also been found to lead to a depletion of arachidonoyl‐PI, essential for the synthesis of key phosphoinositides involved in intracellular signalling [102, 103]. Moreover, loss of MBOAT7/LPIAT1 function is often associated with decreased membrane integrity and fluidity, resulting from PI alterations. MBOAT7/LPIAT1‐mediated acylation of polyunsaturated fatty acids (PUFAs) into PI ensures that the lipid membrane remains flexible and capable of dynamic signalling responses [88]. In an MBOAT7/LPIAT1 deficient state, an increase in monounsaturated and saturated fatty acids, such as oleic acid and palmitic acid in PI occurs, resulting in stiffer and less fluid membranes. Metabolically, these alterations have profound consequences for insulin sensitivity and metabolic homeostasis, resulting from changes to membrane‐associated signalling cascades due to deficiencies in MBOAT7/LPIAT1 activity. Secondly, the downregulation of MBOAT7 has been found to be a driver of MAFLD/MASH progression via the LPI/GPR55 pathway [47, 100]. That is, MBOAT7 primarily converts LPI to PI through acylation, reducing circulating levels of LPI. Lastly, MBOAT7 downregulation has been associated with increased *de novo* lipogenesis and hepatic steatosis.

## 6 MBOAT7/LPIAT1 and SREBP‐1c as a Driver of De Novo Lipogenesis

Downregulation of MBOAT7/LPIAT1 causes downstream activation of SREBP‐1c, a key transcription factor associated with fatty acid synthesis regulation. In turn, this causes an increase in the expression of lipogenic enzymes such as FAS and ACC, disrupting lipid homeostasis [104, 105]. Stemming from the disruptions to membrane fluidity and PI‐dependent signalling, this compositional shift affects pathways such as the phosphatidylinositol 3‐Kinase/Protein Kinase B/Mammalian Target of Rapamycin (PI3K/AKT/mTOR pathway), resulting in changes to SREBP‐1c activation [97]. In the liver, SREBP‐1c is responsible for lipid production regulation and exportation into the plasma as lipoproteins, while also exporting lipids into the biliary duct as micelles [105]. Furthermore, in the context of metabolic dysfunction, SREBP‐1c also influences the metabolism of carbohydrates, further exacerbating glucose‐related metabolic disorders such as T2D by modulating the expression of genes involved in glycolysis and gluconeogenesis [106]. Undeniably, the link between obesity, T2D and MAFLD highlights the importance of insulin and lipid metabolism dysfunction. Dysfunction of MBOAT7/LPIAT1 leads to elevated levels of lipid accumulation in the liver, contributing to insulin resistance and leading to further downstream detriments to metabolic health.

## 7 MBOAT7/LPIAT1 and Extrahepatic Diseases

The rs641738 C>T single nucleotide polymorphism in MBOAT7 has been associated with increased hepatic fat accumulation and higher risk of hepatocellular carcinoma (HCC) development, particularly in individuals with MAFLD/MASH [107, 108]. Recent bioinformatic analyses utilising various databases have demonstrated that MBOAT7 is significantly overexpressed in HCC tissues, and that the overexpression can be correlated to the tumour stage and survival outcomes [107]. In the brain, mutations to MBOAT7 have been implicated in autosomal recessive intellectual development disorders, such as those seen in macrocephaly, intellectual disabilities and epilepsy [109, 110]. Recent studies have also identified the role of MBOAT7 in the pathogenesis of non‐small cell lung cancer, demonstrating that MBOAT7 expression is upregulated in cell models [111]. Similarly, Phadnis et al. reported that the Golgi‐resident scaffold protein monocyte to macrophage differentiation‐associated promoted susceptibility to ferroptosis in ovarian and renal carcinoma cells in an MBOAT7‐dependent manner [112].

## 8 Therapeutic Strategies Targeting LPI/GPR55 Axis

While improvements in general metabolic health may contribute to hepatic improvement, the risk–benefit of pharmacological treatment still favours a liver‐directed therapeutic strategy involving disruption of the LPI/GPR55 axis. In both human and mouse models of MASLD, it is observed that LPI/GPR55 activation is upregulated, driving hepatic lipogenesis and HSC activation, leading to the progression of fibrosis [47]. Additionally, advancements in biotechnology, including the use of targeted nanoparticles and related liver‐specific nanoplatforms, increase the feasibility of inducing localised antagonism of the LPI/GPR55 axis [113, 114, 115]. Nevertheless, systemic modulation may also contribute to overall hepatic improvement. Visceral white adipose tissue (WAT) shows higher GPR55 expression and circulating LPI levels, and antagonistic action may reduce lipogenic gene expressions [50]. Also, by disrupting the LPI/GPR55 axis in pancreatic β cells, this encourages other ligands to bind to GPR55, showing an enhanced glucose‐stimulated insulin secretion [116, 117].

The growing understanding of the LPI/GPR55 axis and its relation to the pathophysiology of MAFLD has positioned it as a key therapeutic target. Despite investigations into the effectiveness of other pharmacological treatments for MASLD, the proposal to target GPR55 in the development of pharmacological therapies for MASLD remains robust. Currently, it is estimated that 30%–40% of all approved pharmacological treatments in clinical practice target the GPCR superfamily, of which GPR55 falls under [118, 119]. Furthermore, targeting GPR55 in MASLD represents an exciting frontier as GPR55 exhibits ligand‐functional selectivity, thus showing high therapeutic potential [120]. For example, coupling of LPI/GPR55 promotes proliferation and migration of cancer cells; while activation of GPR55 with other ligands such as anandamide may trigger cell apoptosis [120, 121]. Moreover, studies using stereoisomers of lysophospholipids found that the functional outcomes of GPR55 may be modulated by different stereoisomers, further reinforcing the idea that GPR55 exhibits structural selective bias [122]. Previous studies have shown that the LPI/GPR55 axis promotes *de novo* lipogenesis via activation of AMP‐activated protein kinase (AMPK) and ACC signalling [47], and pharmacological inhibition of GPR55 using antagonist CID16020046 has shown improvements to lipid accumulation and improved liver histology [69, 123]. Thus, it is stipulated that the liver‐specific pharmacological targeting of GPR55 in the context of MASLD remains highly plausible and may alleviate side effects seen in approved pharmacotherapies. The bioactive lipid LPI has also been identified to be a key driver behind chronic liver diseases [47, 124]. Therapeutic approaches so far have been designed around modulating the activity of GPR55 and focus on small‐molecule agonists and antagonists. However, as previously mentioned, targeting the ECS proves challenging due to unwanted off‐target effects and tissue specificity. Previous investigations into the modulation of GPR55 signalling have focused on agonistic and antagonistic approaches using small molecules and phytocannabinoids, as discussed below.

### 8.1 Small Molecule Agonists/Antagonists of LPI/GPR55

#### 8.1.1 Agonists

GPR55 agonists have been developed to selectively activate GPR55 receptors, with particular focus on their role in various tissues such as the pancreas, where modulating this receptor's activity may influence metabolic and physiological processes (Table S2). Previous evidence suggests that LPI induces insulin release from pancreatic islets, leading to improved glucose metabolism [125]. While the general consensus is that LPI/GPR55 activation in the liver is detrimental, a previous study by Lipin et al. suggests that GPR55 deficiency in mice was associated with increased adiposity and impaired insulin signaling in peripheral metabolic tissues [126]. On the contrary, other studies have shown that the LPI/GPR55 axis has a negative impact on hepatic steatosis and inflammation [47, 48, 127]. Interestingly, a comprehensive survival and prognosis analysis of GPR55 expression in HCC revealed that overexpression of GPR55 played a protective role. This led to better overall survival and prognosis in HCC patients, potentially highlighting the beneficial role of GPR55 in liver cancer prognosis [128]. The specific role of LPI/GPR55 remains to be elucidated. However, it appears to be context‐dependent, with its effects varying based on factors such as diet, MetS and the specific cell types involved. This complexity is further compounded by the tissue‐specific expression and function of GPR55. For instance, in certain organs, such as the pancreas, activation of GPR55 may exert beneficial effects, particularly in the regulation of insulin secretion and glucose homeostasis.

#### 8.1.2 Antagonists

Antagonism of the LPI/GPR55 axis has emerged as a promising strategy to counteract its pro‐inflammatory and metabolically disruptive effects, particularly in the context of liver and metabolic diseases. This therapeutic approach is supported by growing evidence linking GPR55 activation to disease progression in conditions such as MAFLD and MASH. Several GPR55 antagonists, including both synthetic compounds and repurposed phytocannabinoids, have already been identified and are under investigation for their potential to modulate this pathway (Table S2). Preclinical studies have shown that the use of small molecule antagonists such as CID16020046 and ML193 to downregulate the LPI/GPR55 axis attenuates pro‐inflammatory cytokine production and causes a reduction in fibrosis and low‐grade inflammation [129, 130]. Additionally, other small molecule antagonists like AM251 and rimonabant, both CB1 antagonists targeting the eCBome, have demonstrated LPI/GPR55 axis antagonism [131, 132, 133].

### 8.2 Phytocannabinoids

Phytocannabinoids, the bioactive compounds derived from *Cannabis sativa* , have been extensively studied for their therapeutic potential in modulating the ECS [134]. While past research has primarily examined the role of phytocannabinoids on the canonical receptors, emerging evidence suggests that certain phytocannabinoids are also able to modulate non‐canonical receptors like GPR55 and GPR119 [16]. CBD, a non‐psychoactive phytocannabinoid, is one of the most studied cannabinoids in relation to GPR55. The role of CBD as a therapeutic spans across a wide variety of usage, including epilepsy, pain relief and inflammation [135, 136, 137]. In the liver, CBD acts as a negative allosteric modulator of GPR55, thereby inhibiting its activation by LPI [138]. Furthermore, CBD can also act on Kupffer cells to inhibit pro‐inflammatory cytokine production, while also activating downstream targets such as PPAR‐γ to increase insulin sensitivity [139]. Other phytocannabinoids, such as THC and tetrahydrocannabivarin (THCV), have been reported to interact with GPR55, although their effects are more complex due to the associated psychotropic effects [140]. To fully understand the therapeutic potential of phytocannabinoids in MAFLD, further mechanistic studies are required to investigate the specific interactions between phytocannabinoids, GPR55 and MAFLD. Information such as structure–activity relationship and relevant downstream signalling pathways may prove beneficial in designing phytocannabinoid‐based MAFLD treatments [141].

## 9 Therapeutic Potential and Challenges in Endocannabinoidome‐Targeted Drug Development

Strategies for developing drugs targeting the eCBome and specifically hepatic GPR55 should focus on achieving selective modulation while optimising pharmacokinetics and minimising adverse effects. Tissue‐selective drugs, such as those specifically targeting hepatic GPR55, could enhance therapeutic outcomes while minimising potential adverse effects. Advancements in drug delivery methods, such as those seen in nanoparticle‐based delivery systems, remain a promising approach. In addition, developing biased GPR55 modulators that activate or inhibit downstream pathways with great specificity would imply combination therapies designed with complementary pathways, such as by targeting specific anti‐inflammatory pathways and lipid mediators. Currently, there hasn't been any studies examining the effect of combination treatment. However, a strong rationale exists. GPR55 represents a novel therapeutic target with the potential to complement existing pharmacologic strategies for MASLD and MASH. Unlike agents such as *resmetirom* or GLP‐1 receptor agonists that primarily modulate lipid metabolism or insulin sensitivity, GPR55 signalling is implicated in multiple pathogenic pathways, including hepatic inflammation, fibrogenesis and lipid accumulation [47, 67, 69, 126]. Pharmacological modulation of GPR55, either through antagonism or biased agonism, may therefore provide benefits by attenuating hepatic inflammatory signalling, reducing stellate cell activation and improving mitochondrial lipid handling [67].

Integrating GPR55‐targeted agents within the current therapeutic landscape could address key limitations in MASLD management, such as incomplete resolution of hepatic inflammation and fibrosis despite improvements in steatosis and metabolic parameters. Moreover, as GPR55 is expressed in immune cells, hepatocytes and HSCs, its modulation could confer a more pleiotropic effect than metabolic agents alone, potentially enhancing synergy with approved drugs like *resmetirom* or incretin‐based therapies. Nevertheless, the translational development of GPR55 modulators faces challenges, including the need for receptor‐selective ligands, clarification of context‐dependent signalling (pro‐ vs. anti‐inflammatory roles) and safety evaluation in long‐term metabolic settings.

Disruption of the LPI/GPR55 axis in the pancreas will increase the release of insulin, which would further compound the effect seen in GLP‐1R agonists. Furthermore, co‐administration of GPR55 antagonists with farnesoid X receptor (FXR) agonists could provide complimentary gut‐barrier benefits, as well as improved immune function [142]. Combining GPR55‐targeting agents with GLP‐1 receptor agonists or FXR agonists offers a promising strategy. GPR55 modulation can complement GLP‐1 receptor agonists by enhancing insulin sensitivity and reducing hepatic lipid accumulation, while also synergizing with FXR agonists to further regulate bile acid metabolism, inflammation, and fibrosis. This multi‐targeted approach could yield enhanced efficacy through parallel modulation of metabolic, inflammatory, and fibrotic pathways. Additionally, targeting the LPI/GPR55 axis by inhibiting enzymes involved in LPI synthesis, such as phospholipase A, presents a novel therapeutic approach for MASH. Enzyme inhibitors, particularly those blocking cytosolic phospholipase A_2_, could reduce LPI levels, thereby dampening GPR55‐mediated pro‐inflammatory, fibrotic and metabolic signaling in the liver. This strategy aims to curb hepatic inflammation, fibrosis and lipotoxicity by lowering endogenous LPI production, potentially enhancing the efficacy of combination regimens with GLP‐1R or FXR agonists. Furthermore, by concurrently targeting other components of the endocannabinoid system, such as through GPR119 agonism, we propose that this approach could produce more robust and effective outcomes [143, 144, 145]. Pairing GPR55 with GPR119 agonism can address the previous shortcomings, as a two‐axis strategy involving targeting both the gut‐incretin and hepatic lipid accumulation may prove beneficial.

Although GPR119 agonists have not demonstrated significant efficacy as monotherapy in clinical trials, combining GPR119 activation with GPR55 antagonism may produce complementary effects on metabolic and hepatic pathways. GPR55 antagonism reduces hepatic lipogenesis, inflammation and stellate cell activation, while GPR119 agonism enhances incretin‐mediated insulin secretion and systemic metabolic regulation. Preclinical evidence suggests that simultaneous targeting of these receptors could yield synergistic benefits, particularly when delivered via liver‐directed strategies to minimise off‐target effects and maximise hepatic efficacy.

Despite its promise, harnessing the therapeutic potential of the eCBome presents significant challenges, particularly due to its complex and widespread signalling network. Off‐target effects remain one of the biggest obstacles to successful therapeutic strategies in the eCBome. However, in the context of GPR55 and MAFLD, additional obstacles complicate this approach. For one, the context‐dependent nature of GPR55 signalling complicates successful therapeutic targeting. Activation of GPR55 in Kupffer cells may cause pro‐inflammatory effects, while at the same time causing anti‐inflammatory effects in other macrophages. Besides, GPR55 interacts with other signalling pathways such as the ECS and inflammatory mediators, underscoring the need for mechanistic studies to identify these interactions to avoid unwanted modulation of downstream signalling pathways.

## 10 Conclusion

The LPI/GPR55 axis, in addition to MBOAT7/LPIAT1 activity, represents a key mechanistic link between dysregulated lipid signalling and disease pathogenesis. Accumulating evidence suggests that the LPI/GPR55 axis modulates key hepatic pathological processes, including hepatic lipogenesis, chronic low‐grade inflammation and fibrogenesis. Despite its therapeutic potential, several unresolved challenges impede drug development. One such major barrier is the context‐dependent signalling role of GPR55, which exerts microenvironment‐specific and even a dichotomous effect across metabolic tissues. LPI‐induced activation of GPR55 in the liver activates Kupffer cells, promoting a pro‐inflammatory M1 phenotype, leading to activation of inflammatory pathways, while activation of GPR55 in pancreatic β‐cells has been associated with improved insulin secretion and sensitivity. Therefore, a lack of tissue‐selective drug delivery methods risks off‐target effects, especially given the receptor's widespread distribution in the brain, immune cells, adipocytes and pancreatic islets. Indeed, targeting the eCBome in the context of MAFLD/MASH is novel, especially given that most prior studies on the LPI/GPR55 axis have omitted the clear biochemical role of MBOAT7/LPIAT1. Specifically, prior studies have examined the pathway‐specific roles of GPR55 in hepatocytes, Kupffer cells and HSCs cells, and the role of LPI as a signalling lipid, while MBOAT7/LPIAT1 studies have focused on the genetic and functional aspects of MBOAT7/LPIAT1 downregulation in membrane rigidity and lipogenesis. This work presents a conceptual framework, by intersecting the activity of MBOAT7/LPIAT1 and the LPI/GPR55 axis, to fill the gap in knowledge by proposing that MBOAT7/LPIAT1 acts as an upstream regulator of LPI levels, thereby influencing GPR55 activation in the liver. Furthermore, the downregulation of MBOAT7/LPIAT1 because of metabolic dysfunction drives, in turn, pathological GPR55 activity. In the absence of comprehensive investigations establishing the MBOAT7‐GPR55 linkage in MAFLD‐specific models using both lipidomic profiling and functional receptor assays, we propose an integrative framework that connects lipid metabolism and GPR55 signalling dynamics with receptor pharmacology in the context of MAFLD pathogenesis.

Future studies should examine the effectiveness of a combination therapy of the LPI/GPR55 axis and MBOAT7/LPIAT1 modulation through gene therapy, such as gene restoration and a pharmacological approach. By integrating the role of MBOAT7/LPIAT1 function into the pathological framework of LPI/GPR55 signalling in MAFLD, we offer a novel perspective that advances the current understanding of lipid‐mediated signalling in MAFLD/MASH. Bridging these two mechanistic pathways provides a more integrated view of the interplay between metabolic and immune dysfunction, underscoring the potential for dual‐targeted therapeutic strategies. In doing so, this review sets a new conceptual precedent in the field, establishing a foundation for future experimental and translational work into drug development, examining the crosstalk between MBOAT7 and GPR55 as a central axis in MAFLD/MASH pathophysiology.

## Funding

M.F. and M.P. received funding from LIPOVEXA S.r.l. External funders had no influence on the content of the manuscript.

## Conflicts of Interest

M.F. is an inventor of a patent related to oleoyl‐LPI mimetics. M.F. is a member of LIPOVEXA S.r.l., a spin‐off company focused on developing innovative treatments for diabetes, obesity and liver health. M.P. received a stipend from LIPOVEXA S.r.l. The other authors declare no conflicts of interest.

## Supporting information


**Table S1:** Summary of key studies investigating LPI–GPR55 modulation in hepatic steatosis, inflammation, and fibrosis. It summarises published studies evaluating LPI/GPR55 modulation in hepatic models. Human investigations to date are observational, quantifying endogenous LPI and GPR55 expression. In mice, both endogenous (diet‐ or toxin‐induced) and exogenous (LPI or synthetic ligand administration) models demonstrate that GPR55 activation promotes hepatic lipid accumulation and fibrogenic signalling. Exogenous LPI is typically administered intravenously at 0.5 mg/kg in vivo and 1–10 μM in vitro, while GPR55 agonist O‐1602 and antagonist CID16020046 are commonly tested at ~1 mg/kg intraperitoneally in rodent models. Sex differences have not been systematically evaluated, most animal studies use male mice, and human cohorts report mixed‐sex populations without stratified analysis. This gap highlights the need for future investigations incorporating sex as a biological variable in LPI–GPR55 pathway research.

**Table S2:** Pharmacological agents targeting GPR55.

## Data Availability

Data sharing not applicable to this article as no datasets were generated or analysed during the current study.
