# Supplementary material for: Targeting the LPI/GPR55 Axis in MAFLD and MASH: Novel Insights, Therapeutic Strategies and Future Directions
Source: Liver Int. 2026 Mar 13;46(4):e70576. doi: 10.1111/liv.70576 (PMC12983196; doi:10.1111/liv.70576)
Supplement: Supplementary file 1 — Table S1: Summary of key studies investigating LPI–GPR55 modulation in hepatic steatosis, inflammation, and fibrosis. It summarises published studies evaluating LPI/GPR55 modulation in hepatic models. Human investigations to date are observational, quantifying endogenous LPI and GPR55 expression. In mice, both endogenous (diet‐ or toxin‐induced) and exogenous (LPI or synthetic ligand administration) models demonstrate that GPR55 activation promotes hepatic lipid accumulation and fibrogenic signalling. Exogenous LPI is typically administered intravenously at 0.5 mg/kg in vivo and 1–10 μM in vitro, while GPR55 agonist O‐1602 and antagonist CID16020046 are commonly tested at ~1 mg/kg intraperitoneally in rodent models. Sex differences have not been systematically evaluated, most animal studies use male mice, and human cohorts report mixed‐sex populations without stratified analysis. This gap highlights the need for future investigations incorporating sex as a biological variable in LPI–GPR55 pathway research. [file LIV-46-0-s002.docx]

**Supplementary Table 1. Summary of key studies investigating LPI–GPR55 modulation in hepatic steatosis, inflammation, and fibrosis**

| **Study** | **Species / Model** | **Experimental Design** | **LPI or Ligand Type** | **Route / Dose / Concentration** | **Endogenous or Exogenous** | **Sex Reported / Analysed** | **Key Findings** |
| --- | --- | --- | --- | --- | --- | --- | --- |
| **Fondevila et al., 2020 (*Hepatology)*** | Human liver tissue (NAFLD/NASH) | Cross-sectional analysis of hepatic GPR55 and plasma LPI levels | Endogenous LPI | N/A | Endo | Mixed cohort (sex distribution reported but not analysed separately) | Hepatic GPR55 expression and plasma LPI elevated in NASH; correlated with steatosis and fibrosis severity |
| **Fondevila et al., 2020 (*Hepatology)*** | C57BL/6J mice (HFD, MCD, and CCl₄ models) | GPR55 knockdown and exogenous LPI administration | L-α-LPI | i.v. 0.5 mg kg^-1^ daily ×7 days | Exo | Not specified (male-only implied) | LPI promoted steatosis, inflammation, and fibrosis; GPR55 silencing protected against diet-induced NASH |
| **Fondevila et al., 2020 (*Hepatology)*** | THLE2, HepG2, LX-2 cells | LPI treatment ± GPR55 silencing | L-α-LPI | 1–10 µM for 24 h | Exo | N/A | Increased lipid accumulation and stellate cell activation via AMPK–ACC and ERK pathways |
| **Kim et al., 2021  *(Int J Mol Sci)*** | C57BL/6J mice (HFD) | GPR55 agonism/antagonism in vivo | O-1602 (agonist); CID16020046 (antagonist) | O-1602: 1 mg kg^-1^ i.p. daily; CID16020046: 1 mg kg^-1^ i.p. daily ×8 weeks | Exo | Not specified (likely male) | O-1602 enhanced hepatic steatosis; GPR55 blockade attenuated lipid accumulation and insulin resistance |
| **Kim et al., 2021 *(Int J Mol Sci)*** | HepG2 and primary mouse hepatocytes | O-1602 exposure | O-1602 (GPR55 agonist) | 5–10 µM for 24 h | Exo | N/A | Activated PI3K/Akt/SREBP-1c signalling; increased intracellular lipid droplets |
| **Lipina et al., 2018 (*Biochem J)*** | GPR55 knockout mice | Global GPR55 deficiency under chow and HFD feeding | N/A (genetic loss-of-function) | N/A | Endo | Both sexes studied; no liver sex-specific analysis | GPR55⁻/⁻ mice had increased adiposity and impaired insulin signalling; metabolic link to hepatic lipid handling |
| **Shi et al., 2024 (J Med Chem)** | C57BL/6J mice (CCL_4_^-^ and MCD-diet induced fibrosis) | Cyclic peptide antagonist of GPR55 (P1-1) | P1-1 (cyclic peptide) | i.v/i.p 1–3 mg/kg i.p. or s.c., 2–4 weeks | Exo |  | P1-1 rapidly reduced collagen secretion in HSCs, attenuated ROS, improved liver inflammation and hepatocyte apoptosis. |

Supplementary Table 1 summarizes published studies evaluating LPI/GPR55 modulation in hepatic models. Human investigations to date are observational, quantifying endogenous LPI and GPR55 expression. In mice, both endogenous (diet- or toxin-induced) and exogenous (LPI or synthetic ligand administration) models demonstrate that GPR55 activation promotes hepatic lipid accumulation and fibrogenic signalling. Exogenous LPI is typically administered intravenously at 0.5 mg/kg in vivo and 1–10 µM in vitro, while GPR55 agonist O-1602 and antagonist CID16020046 are commonly tested at ~1 mg/kg intraperitoneally in rodent models. Sex differences have not been systematically evaluated, most animal studies use male mice, and human cohorts report mixed-sex populations without stratified analysis. This gap highlights the need for future investigations incorporating sex as a biological variable in LPI–GPR55 pathway research.
