# Supplementary material for: Targeting the LPI/GPR55 Axis in MAFLD and MASH: Novel Insights, Therapeutic Strategies and Future Directions
Source: Liver Int. 2026 Mar 13;46(4):e70576. doi: 10.1111/liv.70576 (PMC12983196; doi:10.1111/liv.70576)
Supplement: Supplementary file 2 — Table S2: Pharmacological agents targeting GPR55. [file LIV-46-0-s001.docx]

| Compound name | Mechanism | Development status | Disease | Outcome |
| --- | --- | --- | --- | --- |
| CID16020046 | Antagonist | Preclinical | Inflammatory conditions, MAFLD/MASH | Reduced lipid accumulation and serum triglyceride levels |
| O-1602 | Agonist | Preclinical | Neurological disorders, MAFLD/MASH | Induced hepatic steatosis via SREBP-1c pathway |
| ML-193 | Antagonist | Preclinical | Parkinson’s | Improved motor deficits |
| ETX-018810 | Antagonist | Phase 2 | Neuropathic pain | Trial completed  (NCT04688671) |
| KLS-13019 | Antagonist | Preclinical | Neuropathy | Prevent neuron damage |
| P1-1 | Antagonist | Preclinical | Liver inflammation/fibrosis | Reduced inflammation and fibrosis |
| LPI | Agonist | Preclinical | MAFLD/MASH | Lipid accumulation |

**Supplementary Table 2. Pharmacological agents targeting GPR55.**
